# Supplementary material for: Effects of high-intensity respiratory muscle training on respiratory muscle strength in individuals with Parkinson’s disease: Protocol of a randomized clinical trial
Source: PLoS One. 2023 Sep 8;18(9):e0291051. doi: 10.1371/journal.pone.0291051 (PMC10490961; doi:10.1371/journal.pone.0291051)
Supplement: S1 File — (PDF) [file pone.0291051.s001.pdf]

EFFECTS OF HIGH-INTENSITY RESPIRATORY MUSCLE TRAINING ON RESPIRATORY MUSCLE STRENGTH IN INDIVIDUALS WITH PARKINSON'S DISEASE: PROTOCOL OF A RANDOMIZED CLINICAL TRIAL

Coordinating researcher: Prof. Christina Danielli Coelho de Morais Faria, PT, Ph.D., professor of the Department of Physiotherapy, School of Physical Education, Physiotherapy and Occupational Therapy, Universidade Federal de Minas Gerais.

Area of knowledge: Rehabilitation Sciences

Belo Horizonte

Universidade Federal de Minas Gerais

School of Physical Education, Physiotherapy, and Occupational Therapy

2021

ABSTRACT:

Background: Individuals with PD commonly have a significant reduction in respiratory muscle strength and inspiratory muscle endurance, and it can intensify with the disease progression. Respiratory muscle training has been shown to increase respiratory muscle strength in individuals with Parkinson's disease (PD). However, the effect size on other functional outcomes has not been determined and/or investigated. In addition, studies investigating the effects of high-intensity respiratory muscle training (inspiratory and expiratory) in this population were not found.

Objectives: To investigate the efficacy of high-intensity respiratory muscle training (combined inspiratory and expiratory muscle training) in improving inspiratory and expiratory muscle strength, inspiratory muscle endurance, peak cough flow, dyspnea, fatigue, exercise capacity, and quality of life in this population.

Design: A randomized controlled trial with blinded evaluation will assign eligible participants to either high-intensity respiratory muscle training (experimental group) or sham training (control group). Individuals will perform a home-based intervention, not directly supervised, consisting of two daily 20-min sessions (morning and afternoon), seven times a week, during eight weeks. Study Outcomes: Primary outcomes are inspiratory and expiratory muscle strength. Secondary outcomes are respiratory muscle endurance, peak cough flow, dyspnea, fatigue, exercise capacity, and quality of life. The outcomes will be measured at baseline, post-intervention (after the 8-week intervention), and one month after the cessation of the intervention (12-week follow-up), by trained evaluators.

Conclusion: The results of this trial will provide valuable new information on the effects of high-intensity respiratory muscle training in improving muscle strength and other

functional outcomes in individuals with PD.

1.0. Introduction and Literature Review

1.1. Parkinson's Disease

Neurological diseases are the leading cause of disability worldwide (GROUP, 2015). Among the diseases analyzed by the Global Burden of diseases, injuries, and risk factors study, Parkinson's disease (PD) was the one with the highest growth in prevalence, disabilities, and deaths (GROUP, 2015). From 1990 to 2016, there was a 2.4-fold increase in the number of people with PD (DORSEY et al., 2007). PD is the second most prevalent neurodegenerative disease globally. It is estimated that approximately 6.1 million people have PD worldwide (DORSEY et al., 2016). However, these numbers may be underestimated, since there are few official data on the incidence and prevalence of PD in many countries, especially underdeveloped and developing ones (DORSEY et al., 2016), such as Brazil.

A study carried out in a Brazilian city showed a prevalence of 3.3% of PD among older adults (BARBOSA et al. 2006). According to statistics, there are approximately 30 million older adults in Brazil (CONTINUA, 2020). If this prevalence is extrapolated to the older population in Brazil, it is projected that approximately one million Brazilians have PD. Furthermore, estimates indicate that the incidence of PD will approximately double by 2030 (DORSEY et al., 2007).

PD is a degenerative and progressive disease of the central nervous system that causes motor and non-motor disabilities (KALIA et al., 2015). The neuropathology of PD is multifactorial and heterogeneous, but there are some pathological hallmarks (MCGREGOR et al., 2019). A neuropathological hallmark is the presence of intracellular inclusions resulting from the aggregation of the α-synuclein protein, called Lewy bodies (KALIA et al., 2015). The most well-known site of Lewy body deposition and neurodegeneration is the midbrain, specifically the dopaminergic neurons of the

substantia nigra pars compacta (MCGREGOR et al., 2019). This leads to loss of dopamine in the nigrostriatal system (KALIA et al., 2015). Finally, other non-dopaminergic systems are also affected by disease progression, such as cholinergic, serotonergic, and noradrenergic projections (MCGREGOR et al., 2019; SCHAPIRA et al., 2017).

PD has four classic motor signs (bradykinesia, rigidity, postural instability, and resting tremor), called cardinal signs (KALIA et al., 2015; MORRIS et al., 2000; SCHEKMAN et al., 2001; SHULMAN et al., 2011). In addition, PD causes various non-motor disabilities as well, such as ventilatory and respiratory disorders, cognitive deficits, fatigue, autonomic dysfunction, sleep disturbances, and gastrointestinal disorders (KALIA et al., 2015).

The International Classification of Functioning, Disability, and Health (ICF) (ORGANIZAÇÃO MUNDIAL DE SAÚDE, 2003) provides an important framework for describing functionality, guiding therapeutic goal setting and intervention plans. In the study by Raggi et al. (2011), individuals with PD reported impairment in body structures and functions, activity limitations, and restrictions on social participation (RAGGI et al. 2011). In addition, environmental factors were qualified as barriers or facilitators, such as attitudes of family members and policies of social security services (RAGGI et al. 2011).

1.2. Respiratory Disorders in Parkinson's Disease

Respiratory and ventilatory disorders are non-motor signs and symptoms commonly observed in individuals with PD. According to studies, individuals with PD commonly have respiratory muscle weakness (HAAS et al., 2004; GUEDES et al., 2012; PAL et al., 2007; SANTOS et al. 2019), reduced lung function, altered kinematics of the

rib cage, reduced laryngeal sensitivity, and compromised cough reflex (EBIHARA et al., 2003; FONTANA et al., 1998; PITTS et al., 2008). The prevalence of respiratory disorders in PD is still underestimated. In a literature review, it was reported that the prevalence of restrictive disorders in individuals with PD ranged from 28% to 94%, and obstructive disorders ranged from 6.7% to 67% (D'ARRIGO et al., 2020).

The pathophysiology of respiratory disorders in PD is still uncertain. Biomechanical characteristics of PD may be related to the respiratory disorders found in these individuals, such as postural changes and stiffness in the thoracic muscles, reduced thoracic mobility (CARDOSO et al., 2002), changes in the activation and coordination of the upper airway muscles (MONTEIRO et al., 2014). In addition, tremors (BROWN et al., 1997) and bradykinesia can compromise the respiratory function in individuals with PD (CARDOSO et al., 2002; GUEDES et al., 2012).

Neuropathological changes have also been associated with these respiratory disorders. As previously described, one of the neuropathological characteristics of PD is the depletion of dopaminergic neurons in the substantia nigra of the midbrain. This neuronal destruction also happens in the brain, as in the brainstem nuclei that control sleep and breathing. In addition, aggregation of the α-synuclein protein occurs first in the medulla, and later in the respiratory centers (BRAAK H., 2003; DOCU AXELERAD et al., 2021).

It has been widely described in the scientific literature that individuals with PD have reduced respiratory muscle strength when compared to healthy individuals of the same sex and age. Guedes et al. (2012) found maximum inspiratory pressure (MIP) and maximum expiratory pressure (MEP) values significantly lower in individuals with PD (MIP (men)= 65.0±17.9cmH_2O; MIP (women)= 55.6±18.8cmH_2O; MEP (men)= 81.9±14.7cmH_2O; MEP women= 64.4±21.9cmH2O) when compared to healthy

individuals of the same sex and age (MIP (men)= 86.9±6.2cmH2O; MIP (women)= 70.0±14.1cmH2O; MEP (men)= 120.0 ±29.4cmH2O; MEP (women)= 105.6±19.4cmH2O) (GUEDES et al. 2012).

Studies have already shown that the reduction in respiratory muscle strength is present in the early stages of PD (BAILLE et al., 2019) and that it intensifies with the progression of the disease (SANTOS et al. 2019). Although respiratory disorders are frequent and potentially serious, it may not be observed early due to the reduced level of physical activity of individuals with PD (HAAS et al., 2004; HOVESTADT et al., 1989; SATHYAPRABHA et al., 2005). Due to the reduced level of physical activity, these individuals may not perform activities that require sufficient energy expenditure to manifest signs of respiratory dysfunction. (VAN NIMWEGEN et al., 2011). Therefore, interventions to improve respiratory muscle strength are commonly initiated in advanced stages.

As previously described, individuals with PD may have reduced muscle strength in both inspiratory and expiratory groups. Both muscle groups are essential for maintaining adequate respiratory function. In individuals with PD, it has already been observed that inspiratory muscle weakness predisposes to the onset of atelectasis and secretion retention, which is an important risk factor for infections (SAPIENZA et al., 2003). In expiratory muscle weakness, there is an increased risk of accumulation of secretions and the development of respiratory complications, such as pneumonia (BACKSTROM et al., 2018). Therefore, both inspiratory and expiratory muscle strength should be measured in individuals with PD and, when muscle weakness is observed, interventions to increase muscle strength of both muscle groups should be performed.

1.3. Respiratory Muscle Training

Several therapeutic interventions have been used to increase respiratory muscle strength in these individuals (HAAS et al. 2004; KILLIAN et al., 1984; MCMAHON et al. 2020; SANTOS et al. 2011). A systematic review with meta-analysis assessed the effects of non-pharmacological interventions on the respiratory function of individuals with PD (MCMAHON et al. 2020). In this systematic review, studies that assessed the effects of several kinds of interventions were included, such as, such as aerobic exercise, yoga, cycling, peripheral muscle strengthening, and specific strengthening of the respiratory muscles (inspiratory and expiratory muscle training) (MCMAHON et al. 2020).

The results of this meta-analysis demonstrated that global exercises and respiratory muscle training had a statistically significant increase in respiratory muscle strength, peak expiratory flow, and respiratory muscle endurance (MCMAHON et al. 2020). In addition, respiratory muscle training significantly increased peak cough flow and dyspnea, but it was not possible to perform a meta-analysis for these outcomes due to data heterogeneity, (MCMAHON et al. 2020).

Respiratory muscle training has the potential to improve respiratory function (MCMAHON et al. 2020). Systematic reviews with meta-analysis have already demonstrated that respiratory muscle training is effective in increasing the respiratory function of individuals with different health conditions, such as individuals with chronic obstructive pulmonary disease (SHAFFER et al., 1995), heart failure (MCELFRESH et al., 2012), stroke (MENEZES et al., 2016) and in mechanical ventilation (ELKINS et al., 2015). In individuals with PD, a systematic review of randomized controlled trials (RCT) investigated the effects of respiratory muscle strengthening on respiratory function (RODRÍGUEZ et al. 2020). Only five studies from three different RCTs were included. Of these five studies, one investigated the effect of inspiratory muscle training on

inspiratory muscle strength, respiratory muscle endurance, reduction of dyspnea, and quality of life (INZELBERG et al. 2005). Two studies from a single RCT evaluated the effect of expiratory muscle strengthening on swallowing (TROCHE et al., 2010), expiratory muscle strength, and lung function (SAPIENZA et al., 2011). In addition, in two other studies from a single RCT, the effectiveness of respiratory muscle training at peak cough flow, inspiratory and expiratory muscle strength (REYES et al., 2018), and phonatory parameters (REYES et al., 2019). Finally, in two other studies from a single RCT, the effects of respiratory muscle training on peak cough flow, inspiratory and expiratory muscle strength (REYES et al., 2018), and phonatory parameters (REYES et al., 2019) were investigated.

In those previous studies, the training load ranged from 15% to 60% of MIP (INZELBERG et al. 2005) and ranged from 50% to 75% of MEP (REYES et al. 2018; REYES et al. 2019; SAPIENZA et al. 2011; TROCHE et al. 2010), duration ranged from four (SAPIENZA et al. 2011; TROCHE et al. 2010) to 12 weeks (INZELBERG et al. 2005) and frequency from five to six times a week (INZELBERG et al. 2005; REYES et al.2018; REYES et al. 2019). It was not possible to evaluate the superiority of one protocol over the other due to heterogeneity in outcome measures. The systematic review demonstrated that respiratory muscle training is effective in improving respiratory muscle strength and endurance, lung function, swallowing, dyspnea, and phonatory parameters in individuals with PD (RODRÍGUEZ et al., 2020). However, a small number of studies were found (only three RCTs). In addition, it has been shown that existing studies have moderate methodological quality and heterogeneity in their outcome measures (RODRÍGUEZ et al., 2020). Therefore, the authors concluded that the evidence is limited for any recommendation for clinical practice, suggesting the execution of other RCTs (RODRÍGUEZ et al., 2020).

In these studies, an increase in MIP was found, ranging from -1.28cmH_2O (INZELBERG et al., 2005) to -16cmH_2O (REYES et al., 2018). In addition, increases in MEP from 15.5cmH_2O (REYES et al., 2018) to 27.97cmH_2O (SAPIENZA et al., 2011) were observed when expiratory muscle training was performed. In other studies that investigated the effect of high-intensity respiratory muscle training in other populations, an improvement in MIP of -43cmH_2O and MEP of 51cmH_2O was found, that is, approximately double (GOMES NETO et al., 2018; HILL et al., 2006; PARREIRAS DE MENEZES et al., 2019). In addition, the benefits of respiratory muscle training on other functional outcomes (perception of dyspnea, fatigue, and exercise capacity) seem to be obtained when a high training volume (load, duration, and frequency) is applied (GOMES NETO et al., 2018; HILL et al., 2006). Therefore, the effects of high-intensity respiratory muscle training of higher intensities (load, intensity, and volume) still need to be investigated in individuals with PD.

Studies investigating the effects of respiratory muscle training on fatigue, and exercise capacity in individuals with PD were not found. In individuals with other neurological diseases, respiratory muscle training has been shown to improve performance in activities of daily living (CHEN et al., 2016), exercise capacity (SUTBEYAZ et al., 2010), and quality of life (SUTBEYAZ et al., 2010). In individuals with PD, respiratory muscle weakness showed a statistically significant correlation with exercise capacity (HAAS et al., 2004). Therefore, respiratory muscle training can improve these important outcomes in this population.

Finally, studies investigating the combination of these training (inspiratory plus expiratory) in this population were not found. Performing combined inspiratory and expiratory muscle training using a single piece of equipment is cheaper and feasible, takes less time, and is easy to use. Thus, it is important to investigate the effects of t high-

intensity respiratory muscle training (combined inspiratory and expiratory muscle training) on inspiratory and expiratory muscle strength, inspiratory muscle endurance, peak cough flow, dyspnea, fatigue, exercise capacity, and quality of life in this population.

2.0.OBJECTIVES

- Primary objective: To investigate the effects of high-intensity respiratory muscle training (combined inspiratory and expiratory muscle training) on inspiratory and expiratory muscle strength in individuals with PD.
- Secondary objective: To investigate the effects of high-intensity respiratory muscle training (combined inspiratory and expiratory muscle training) in improving inspiratory muscle endurance, peak cough flow, dyspnea, fatigue, exercise capacity, and quality of life in this population.

3.0.METHODS

A prospective, superiority parallel-group randomized controlled trial, with concealed allocation, allocation ratio 1:1, blinded evaluations, and intention-to-treat analysis will be carried out at the Department of Physiotherapy, Universidade Federal de Minas Gerais (UFMG).

The project will be submitted to the Research Ethics Committee of the Universidade Federal de Minas Gerais. All consents for the development of the study were obtained. This trial will be registered at www.ClinicalTrials.gov and performed following the Consolidated Standards of Reporting Trials (CONSORT) (SCHULZ et al. 2010). All individuals will be instructed on the procedures and will sign a statement of informed consent. During the study, measures to prevent the spread of the coronavirus will be observed, such as attendance of only one participant per time, use of mask and alcohol gel (COMITÊ PERMANENTE DE ENFRENTAMENTO DO NOVO

CORONAVÍRUS DA UFMG, 2020; MINISTÉRIO DA SAÚDE, 2020).

3.1. Participants

A non-probabilistic sample will be recruited from the community through contact with health centers, research groups, and university extension programs. Individuals will be included according to the following criteria: Parkinson's disease diagnosed by a neurologist; ability to walk independently, with or without assistive devices; taking antiparkinsonian medication, and who have been medically stable for at least six months; classified between stages 1-3 of the modified Hoehn & Yahr Scale (SCHENKMAN et al., 2001); maximum inspiratory pressure less or equal to -80 cmH_2O or maximum expiratory pressure less or equal to 90 cmH_2O. Individuals will be excluded according to the following criteria: possible cognitive impairment as determined by cutoff scores (in points) of the Mini-Mental Status Examination; use of deep brain stimulation (DBS); smokers or who stopped smoking less than six months ago; have been affected by respiratory or cardiac infections in the last month; had any other neurological, musculoskeletal, cardiovascular or respiratory disorders that could affect their ability to perform the tests.

3.2. Randomization

Participants will be allocated into two groups (experimental and control). The allocation sequence will be generated by a website (www.randomization.com). A research assistant not involved in the recruitment will make the randomization and place the sequence in numbered and sealed opaque envelopes. A trained examiner, blinded to the group allocation sequence, will collect pre-intervention measurements. Afterward, a trained research assistant will reveal the contents of these envelopes.

Some actions will be taken in an attempt to blind the volunteers as to the intervention received/group allocation. At baseline, the individual will not be told the difference between interventions. In addition, all devices will be wrapped with an opaque material, so that the respiratory training load is not visualized. Finally, in the evaluation one month after the end of the intervention (week 12) the individuals will be asked if they suspected which group they were allocated to. The same procedure will be performed with the evaluators. The hit rate will be used as an indirect measure of blinding success.

3.3. Intervention

Participants will undergo high-intensity inspiratory and expiratory muscle training (experimental group), or a placebo intervention. Both groups will do the same exercise protocol, the only difference between the two groups will be the presence/absence of the load imposed during training. The Orygen-Dual Valve device will be used, which provides workloads of up to 70 cmH2O, an adequate seal, through a comfortable and flexible mouthpiece.

Individuals will perform a home-based intervention, split into two daily 20-min sessions (morning and afternoon), totaling 40 min per day, seven times a week, during eight weeks. Each daily session will be composed into four blocks of three minutes, with a two-minute rest between blocks.

To monitor adherence to the protocol, individuals will receive a training diary, in which duration and subjective perception of effort will be recorded using the Borg scale, for each intervention day. If necessary, a caregiver will be instructed to assist the individual in completing the diary.

3.3.1. Experimental Group

The training program will be carried out with the Orygen Dual Valve® (Forumed

S.L., Girona, Spain). Individuals will perform a home-based intervention, split into two daily 20-min sessions (morning and afternoon), totaling 40 min per day, seven times a week, during eight weeks. Each daily session will be composed into four blocks of three minutes, with a two-minute rest between blocks. The initial training load for each participant will be set at 60% of his/her maximal baseline MIP and MEP for both inspiratory and expiratory strength training, respectively. Borg score of dyspnea and effort was also considered for adjusting training intensity, and scores from 4 to 6 were targeted. Once a week, a trained researcher will visit their homes, the MIP and MEP will be measured and the training load will be progressed to ensure that 60% of the new pressure values are maintained.

3.3.2. Control Group

The control group will also perform the exercises using the Orygen Dual Valve® (Forumed S.L., Girona, Spain) device. A sham intervention will be implemented: the initial resistance of the device will be 0cmH2O, and will be maintained throughout the intervention period - there will be no load progression. All procedures adopted with the experimental group, including the weekly home visit, will also be performed with individuals in the control group. However, there will be no real change in the training load. All devices will be wrapped with an opaque material so that the load or possible respiratory training load is not visualized.

3.4. Outcome measurements

Outcome measurements will be performed at baseline (week 0), immediately after training (week 8), and one month after training (week 12). In the initial evaluation, clinical and demographic data of all individuals, identification, and characterization of the sample

will be collected.

In all three evaluations, measurements of MIP and MEP, inspiratory muscle endurance, peak cough flow, dyspnea, fatigue, exercise capacity, and quality of life will be performed. An examiner, blinded to the group allocation, will perform all these measurements. All participants will be instructed not to comment on information about the training received.

3.5. Instruments

3.5.1. Primary Outcomes

3.5.1.1. Inspiratory Muscle Strength

Inspiratory muscle strength will be measured using MIP. Measurements will be performed using a digital manovacuometer (NEPEB-LabCare/UFMG, Brazil) and reported in cmH_2O (PESSOA et al., 2014; PESSOA et al., 2015). The determination of the manovacuometer variables is performed using the MANOVAC 4.0 software. The manovacuometer is the gold standard instrument to measure respiratory muscle strength (PESSOA et al., 2014). Individuals will perform inspirations against an obstructed airway within the mouthpiece. The collection will be performed with the individual sitting in a chair, with the feet and back supported and the torso at a 90-degree angle with the hip. A conventional mouthpiece and nose clip will be used (PESSOA et al., 2015). Individuals will be instructed to breathe comfortably two to three times at tidal volume level. Five acceptable measurements will be taken. The highest pressure of three reproducible measurements, with less than 10% variability, will be recorded and used for analysis (PESSOA et al., 2015).

3.5.1.2. Expiratory Muscle Strength

Expiratory muscle strength will be measured using MEP. MEP will be measured with the same digital manovacuometer (NEPEB-LabCare/UFMG, Brazil), and reported in cmH_2O (PESSOA et al., 2014; PESSOA et al., 2015). To record expiratory pressures, individuals will perform expirations against an obstructed airway within the mouthpiece (PESSOA et al., 2015). The collection protocol will be the same as previously described for the evaluation of inspiratory muscle strength. The highest pressure of three reproducible measurements, with less than 10% variability, will be recorded and used in data analysis (PESSOA et al., 2015).

3.5.2. Secondary Outcomes

3.5.2.1. Inspiratory Muscle Endurance

The number of breaths that the individual can perform will measure inspiratory muscle endurance. Individuals will be instructed to breathe against a submaximal inspiratory load (50% of maximal inspiratory pressure), until fatigue or up to the seven-minute limit (CHARUSUSIN et al., 2013). The POWERbreathe® KH1 instrument will be used, and the recommendations for using the equipment will be followed (CHARUSUSIN et al., 2013). The total number of repetitions will be used for analysis (CHARUSUSIN et al., 2013).

3.5.2.2. Peak Cough Flow

The peak cough flow measurements will be performed with the peak expiratory flow meter (Mini-Wright Peak Expiratory Flow Meter) (FREITAS et al., 2010). For that, the individual must take a deep breath (total lung capacity), and then cough as vigorously as possible (BACH et al., 2006). At least three measurements will be performed that must have a different maximum of 5% between them (FREITAS et al., 2010; PEREIRA et a.,

2002). The highest value, in L/min, will be recorded and used in the analyzes (FREITAS et al., 2010; PEREIRA et al., 2002).

3.5.2.3. Dyspnea

Dyspnea will be measured using the Medical Research Council (MRC) instrument (KOVELIS et al., 2008). The participants will be asked to choose which level best represents how dyspnea limits their activities of daily living on a 5-point scale, in which 0 indicates 'breathless only with strenuous exercise' and 4 indicates 'too breathless to leave the house'. (KOVELIS et al. al., 2008). This measurement tool has adequate measurement properties for evaluating dyspnea in this population (KOVELIS et al., 2008). The total score will be used in the analyses.

3.5.2.4. Fatigue

Fatigue will be measured using the Fatigue Severity Scale (FSS). This instrument measures the impact of fatigue on activities of daily living. Nine statements compose this instrument, and participants will be asked to rate how much they agree with each of the statements. For each item, the score ranges from 1 to 7, where 1 completely disagrees and 7 completely agrees. The FSS is recommended for evaluating fatigue in individuals with PD (FRIEDMAN et al., 2010) and has adequate measurement properties for measuring fatigue in this population (FRIEDMAN et al., 2010; VALDERRAMAS et al., 2012). The total score will be used in the analyzes

3.5.2.5. Exercise Capacity

Exercise capacity will be measured using the six-minute walk test (6MWT) (BLOEM et al., 2016; STEFFEN et al., 2008). The 6MWT will be performed in a 30-meter hallway, delimited by two cones. Individuals will be instructed to walk as far as

possible in six minutes, and standardized stimuli will be provided every minute of the test. Two tests will be performed with an interval of 30 minutes between them, according to the guidelines of the European Respiratory Society / American Thoracic Society (HOLLAND et al., 2014). The test with the greatest distance (in meters) walked will be considered and used in the analyses (HOLLAND et al., 2014).

3.5.2.6. Quality of Life

Quality of life will be measured using the Parkinson's Disease Questionnaire-39 (PDQ-39) (JENKINSON et al., 1997). This instrument consists of 39 items, divided into eight dimensions. For each item, there are five response options, and scores range from 0 (never) to 4 (always or it is impossible for me) (JENKINSON et al., 1997). The score for each domain ranges from 0 (zero) to 100 (one hundred), where zero means better and 100 means the worse quality of life (JENKINSON et al., 1997). The PDQ-39 has adequate measurement properties for measuring fatigue in this population (LANA et al., 2007). The total score will be used for the analysis.

3.6. Sample size calculation

The sample size calculation was performed considering the primary outcome measures (inspiratory and expiratory muscle strength). The effect size for inspiratory muscle training was derived from an RCT with a similar population and intervention. Considering a significance level (α) of 5% and a power of 0.80, thirteen participants per group are required (26 participants). The effect size for expiratory muscle training was also derived from an RCT with a similar population and intervention. Considering a significance level (α) of 5% and a power of 0.80, fourteen participants per group are required (28 participants). Therefore, a sample size of 28 individuals (14 in each group)

was defined (the largest sample size calculated). Assuming an expected dropout rate of 20%, a total sample size of 34 individuals was set (17 in each group).

3.7. Statistical analyzes

All statistical analyzes will be performed by an independent examiner, blinded to the group allocation. Each participant will be assigned with a unique code. All analyses will be performed using SPSS (SPSS Inc., Chicago, IL, USA). The normality of data distribution will be verified for all continuous numeric variables. Descriptive statistics will be calculated for all outcomes. The effects of the interventions will be analyzed from the collected data using intention-to-treat. Data from the last available evaluation will be used for missed sessions. Two-way ANOVA with repeated measures (2*3) will be used to evaluate the differences between groups, considering the time factor (considering baseline, post-intervention, and 4-week follow-up), for the variables: inspiratory and expiratory muscle strength, respiratory muscle endurance, peak cough flow, and exercise capacity. The level of significance will be set at 5% and adjusted for multiple comparisons. Data distribution and equality of variance will also be analyzed, to ensure the parametric analysis has been applied correctly. The effect sizes will be calculated to determine the magnitude of the differences between the groups. The differences between the two mean values will be expressed in units of their SD, expressed as Cohen's d, or mean results for the experimental group minus the mean results for the control group, divided by the SD of the control group. Effect sizes between 0.2 and 0.5 will be considered small; between 0.5 and 0.8, medium; and above 0.8, large.

REFERENCES

BACH, J. R. et al. Expiratory flow maneuvers in patients with neuromuscular diseases. **American journal of physical medicine & rehabilitation**, v. 85, n. 2, p. 105-111, 2006.

BÄCKSTRÖM, D. et al. Early predictors of mortality in parkinsonism and Parkinson disease: A population-based study. **Neurology,** v. 91, n. 22, p. e2045-e2056, 2018.

BAILLE, G. et al. Dyspnea: An underestimated symptom in Parkinson's disease. **Parkinsonism Relat Disord,** v. 60, p. 162-166, 2019.

BARBOSA, M. T. et al. Parkinsonism and Parkinson's disease in the elderly: A community-based survey in Brazil (the Bambuí study). **Movement Disorders,** v. 21, n. 6, p. 800–808, 2006.

BLOEM, B. R. et al. Measurement instruments to assess posture, gait, and balance in Parkinson's disease: Critique and recommendations. **Mov Disord,** v. 31, n. 9, p. 1342-55, 2016.

BORG, G. A. Psychophysical bases of perceived exertion. **Med Sci Sports Exerc,** v. 14, n. 5, p. 377-81, 1982.

BRAAK, H. et al. Staging of brain pathology related to sporadic Parkinson's disease. **Neurobiol Aging,** v. 24, n. 2, p. 197-211, 2003.

BROWN, P.; CORCOS, D. M.; ROTHWELL, J. C. Does parkinsonian action tremor contribute to muscle weakness in Parkinson's disease? **Brain,** v. 120, p. 401-8, 1997.

CARDOSO, S. R.; PEREIRA, J. S. Analysis of breathing function in Parkinson's disease. **Arq Neuropsiquiatr,** v. 60, n. 1, p. 91-5, 2002.

CHARUSUSIN, N. et al. Inspiratory muscle training protocol for patients with chronic obstructive pulmonary disease (IMTCO study): a multicentre randomised controlled trial. **BMJ Open,** v. 3, n. 8, 2013.

CHEN, P.C. et al. Inspiratory muscle training in stroke patients with congestive heart failure: A CONSORT-compliant prospective randomized single-blind controlled trial. **Medicine (Baltimore)**, v. 95, n. 37, p. e4856, 2016.

COMITÊ PERMANENTE DE ENFRENTAMENTO DO NOVO CORONAVÍRUS DA UFMG, 2020. Protocolo de biossegurança e adequação do espaço físico na UFMG. 2020.

CONTINUA, IBGE PNAD. Características gerais dos domicílios e dos moradores 2019. 2020.

D'ARRIGO, A. et al. Respiratory dysfunction in Parkinson's disease: a narrative review. **ERJ Open Research,** v. 6, n. 4, 2020.

DOCU AXELERAD, A. et al. Respiratory Dysfunctions in Parkinson's Disease Patients. **Brain Sciences,** v. 11, n. 5, p. 595, 2021.

DORSEY, E. R. et al. Projected number of people with Parkinson disease in the most populous nations, 2005 through 2030. **Neurology,** v. 68, n. 5, p. 384-6, 2007.

DORSEY, E.R. et al. Global, regional, and national burden of Parkinson's disease, 1990–2016: a systematic analysis for the Global Burden of Disease Study 2016. **The Lancet Neurology,** v. 17, n. 11, p. 939-953, 2018.

EBIHARA, S. et al. Impaired efficacy of cough in patients with Parkinson disease. **Chest,**

v.124, n. 3, p. 1009-15, 2003.

ELKINS M.; DENTICE R. Inspiratory muscle training facilitates weaning from mechanical ventilation among patients in the intensive care unit: a systematic review. **J Physiother,** v. 61, n. 3, p. 125-134, 2015.

FONTANA, G. A. et al. Defective motor control of coughing in Parkinson's disease. **Am J Respir Crit Care Med,** v. 158, n. 2, p. 458-64, 1998.

FREITAS, et al. Relationship between cough strength and functional level in elderly. **Brazilian Journal of Physical Therapy**, v. 14, p. 470-476, 2010.

FRIEDMAN, J. H. et al. Fatigue rating scales critique and recommendations by the Movement Disorders Society task force on rating scales for Parkinson's disease. **Mov Disord,** v. 25, n. 7, p. 805-22, 2010.

GUEDES, L. U. et al. Respiratory changes in Parkinson's disease may be unrelated to dopaminergic dysfunction. **Arq Neuropsiquiatr,** v. 70, n. 11, p. 847-51, 2012.

GROUP, G. N. D. C. Global, regional, and national burden of neurological disorders during 1990-2015: a systematic analysis for the Global Burden of Disease Study 2015. **Lancet Neurol,** v. 16, n. 11, p. 877-897, 2017.

HAAS, B. M. et al. Effects of respiratory muscle weakness on daily living function, quality of life, activity levels, and exercise capacity in mild to moderate Parkinson's disease. **Am J Phys Med Rehabil,** v. 83, n. 8, p. 601-7, 2004.

HOLLAND, A. E. et al. An official European Respiratory Society/American Thoracic Society technical standard: field walking tests in chronic respiratory disease. **Eur Respir**

J, v. 44, n. 6, p. 1428-46, 2014.

HOVESTADT, A. et al. Pulmonary function in Parkinson's disease. **Journal of Neurology, Neurosurgery & Psychiatry,** v. 52, n. 3, p. 329-333, 1989.

INZELBERG, R. et al. Inspiratory muscle training and the perception of dyspnea in Parkinson's disease. **Canadian journal of neurological sciences,** v. 32, n. 2, p. 213-217, 2005.

JENKINSON, C. et al. The Parkinson's Disease Questionnaire (PDQ-39): development and validation of a Parkinson's disease summary index score. **Age and ageing**, v. 26, n. 5, p. 353-357, 1997.

KALIA, L.; LANG, A. Parkinson’s disease. **Lancet [Internet].** 2015; 386 (9996): 896–912.

KILLIAN, K. J. et al. Effect of increased lung volume on perception of breathlessness, effort, and tension. **J Appl Physiol Respir Environ Exerc Physiol,** v. 57, n. 3, p. 686-91, 1984.

KOVELIS, D. et al. Validation of the Modified Pulmonary Functional Status and Dyspnea Questionnaire and the Medical Research Council scale for use in Brazilian patients with chronic obstructive pulmonary disease. **J Bras Pneumol,**v. 34, n. 12, p. 1008-18, 2008.

LANA, R. C. et al. Percepção da qualidade de vida de indivíduos com doença de Parkinson através do PDQ-39. **Brazilian Journal of Physical Therapy**, v. 11, p. 397-402, 2007.

MCELFRESH, J. et al. Inspiratory muscle training in patients with heart failure: a systematic review. **Cardiopulm Phys Ther J.** v. 23, n. 3, p. 29-36, 2012.

MCGREGOR, M. M.; NELSON, A. B. Circuit mechanisms of Parkinson's disease. **Neuron,** v. 101, n. 6, p. 1042-1056, 2019.

MCMAHON, et al. Nonpharmacological Interventions for Respiratory Health in Parkinson's Disease: A Systematic Review and Meta-analysis. **European Journal of Neurology,** 2020.

MENEZES, et al. High-intensity respiratory muscle training improves strength and dyspnea post stroke: a double-blind randomized trial. **Archives of physical medicine and rehabilitation**, v. 100, n. 2, p. 205-212, 2019.

MENEZES, et al. Respiratory muscle training increases strength of respiratory muscles and reduces the occurrence of respiratory complications after stroke: a systematic review. **J Physiother,** v. 62, n. 3, p. 138 -144, 2016.

MINISTÉRIO DA SAÚDE. PORTARIA Nº 1.565, DE 18 DE JUNHO DE 2020. Diário Oficial da União. Ed. 15, sessão 1, p. 64, 2020.

MONTEIRO, L. et al. Swallowing impairment and pulmonary dysfunction in Parkinson's disease: the silent threats. **J Neurol Sci,** v. 339, n. 1-2, p. 149-52, 2014.

MORRIS, M. E. Movement disorders in people with Parkinson disease: a model for physical therapy. **Phys Ther,** v. 80, n. 6, p. 578-97, 2000.

ORGANIZAÇÃO MUNDIAL DE SAÚDE – OMS; ORGANIZAÇÃO PANAMERICANA DE SAÚDE - OPAS. CIF - Classificação Internacional de

Funcionalidade, Incapacidade e Saúde. **São Paulo: Editora da Universidade de São Paulo**, 2003.

PAL, P. K. et al. Pattern of subclinical pulmonary dysfunctions in Parkinson's disease and the effect of levodopa. **Mov Disord,** v. 22, n. 3, p. 420-4, 2007.

PEREIRA, C. A. C. et al. SBPT. Diretrizes para testes de função pulmonar. **J Pneumol**, v. 29, n. 3, p. 207-221, 2002.

PESSOA, I. M. B. S. et al. Test-retest reliability and concurrent validity of a digital manovacuometer. **Fisioterapia e Pesquisa,** v. 21, n. 3, p. 236-242, 2014.

PESSOA, I. M. B. S. et al. Comparison of three protocols for measuring the maximal respiratory pressures. **Fisioterapia em Movimento,** v. 28, n. 1, p. 31- 39, 2015.

PITTS, T. et al. Voluntary cough production and swallow dysfunction in Parkinson's disease. **Dysphagia,** v. 23, n. 3, p. 297-301, 2008.

RAGGI, A. et al. Disability and profiles of functioning of patients with Parkinson's disease described with ICF classification. **International Journal of Rehabilitation Research,** v. 34, n. 2, p. 141-150, 2011.

REYES, A. et al. The effects of respiratory muscle training on peak cough flow in patients with Parkinson's disease: a randomized controlled study. **Clin Rehabil,** v. 32, n. 10, p. 1317- 1327, 2018.

REYES, A. et al. The Effects of Respiratory Muscle Training on Phonatory Measures in Individuals with Parkinson's Disease. **J Voice**, 2019.

RODRÍGUEZ, M. Á. et al. Should respiratory muscle training be part of the treatment of

Parkinson’s disease? A systematic review of randomized controlled trials. **Clinical Rehabilitation,** v. 34, n. 4, p. 429-437, 2020.

SANTOS, R. B. D. et al. Respiratory muscle strength and lung function in the stages of Parkinson's disease. **J Bras Pneumol,** v. 45, n. 6, p. e20180148, 2019.

SAPIENZA, C. et al. Respiratory strength training: concept and intervention outcomes. **Semin Speech Lang,** v. 32, n. 1, p. 21-30, 2011.

SATHYAPRABHA, T. N. et al. Pulmonary functions in Parkinson's disease. **Indian J Chest Dis Allied Sci,** v. 47, n. 4, p. 251-7, 2005.

SCHENKMAN, M. et al. Exercise for people in early- or mid-stage Parkinson disease: a 16-month randomized controlled trial. **Phys Ther**, v. 92, p. 1395- 1410, 2012.

SCHAPIRA, A. H. V. et al. Non-motor features of Parkinson disease. **Nature Reviews Neuroscience,** v. 18, n. 7, p. 435-450, 2017.

SCHULZ, K. F. et al. CONSORT 2010 statement: updated guidelines for reporting parallel group randomised trials. **Trials,** v. 11, n. 1, p. 1-8, 2010.

SHAFFER, T. H. et al. Respiratory muscle function, assessment, and training. **Physical therapy,** v. 61, n. 12, p. 1711-1723, 1981.

SHULMAN, J. M.; DE JAGER, P. L.; FEANY, M. B. Parkinson's disease: genetics and pathogenesis. **Annual Review of Pathology: Mechanisms of Disease,** v. 6, p. 193-222, 2011.

SUTBEYAZ, S.T. et al. Respiratory muscle training improves cardiopulmonary function and exercise tolerance in subjects with subacute stroke: a randomized controlled trial.

Clinical Rehabilitation, v. 24, n. 3, p. 240–250, 2010.

TROCHE, M. S. et al. Aspiration and swallowing in Parkinson disease and rehabilitation with EMST: a randomized trial. **Neurology,** v. 75, n. 21, p. 1912-9, 2010.

VALDERRAMAS, S. et al. Reliability and validity study of a Brazilian- Portuguese version of the fatigue severity scale in Parkinson's disease patients. **Arquivos de neuro-psiquiatria**, v. 70, p. 497-500, 2012.

VAN NIMWEGEN, M. et al. Physical inactivity in Parkinson’s disease. **Journal of neurology**, v. 258, n. 12, p. 2214-2221, 2011.
