# Supplementary material for: Effects of high-intensity respiratory muscle training on respiratory muscle strength in individuals with Parkinson’s disease: Protocol of a randomized clinical trial
Source: PLoS One. 2023 Sep 8;18(9):e0291051. doi: 10.1371/journal.pone.0291051 (PMC10490961; doi:10.1371/journal.pone.0291051)
Supplement: S2 File — (PDF) [file pone.0291051.s002.pdf]

EFEITOS DO TREINAMENTO MUSCULAR RESPIRATÓRIO DE ALTA INTENSIDADE NA FORÇA MUSCULAR RESPIRATÓRIA DE INDIVÍDUOS COM DOENÇADE PARKINSON: PROTOCOLO DE UM ENSAIO CLÍNICO ALEATORIZADO

Pesquisadora coordenadora: Profa. Christina Danielli Coelho de Morais Faria, Ph.D., docente em atividade na UFMG, com vínculo permanente, na unidade Escola de Educação Física, Fisioterapia e Terapia Ocupacional, Departamento de Fisioterapia.

Área de conhecimento: Ciências da Reabilitação

Belo Horizonte Universidade Federal de Minas Gerais

Escola de Educação Física, Fisioterapia e Terapia Ocupacional

Departamento de Fisioterapia

2021

1. INTRODUÇÃO E REVISÃO DA LITERATURA

1.1. Doença de Parkinson

As condições neurológicas são a principal causa de incapacidades mundialmente (GROUP, 2015). Entre as doenças analisadas pelo *Global Burden of diseases, injuries and risk factors study,* a Doença de Parkinson (DP) foi a que apresentou maior crescimento em prevalência, incapacidades e mortes (GROUP, 2015). No período de 1990-2016 houve um aumento de 2,4 vezes no número de pessoas com DP (DORSEY et al., 2007). A DP é asegunda doença neurodegenerativa mais prevalente globalmente. Estima-se que aproximadamente 6,1 milhões de pessoas no mundo tenham DP (DORSEY et al., 2016). Entretanto, esses números podem estar subestimados, uma vez que existem poucos dados oficiais sobre a incidência a prevalência da DP em muitos países, principalmente os subdesenvolvidos e em desenvolvimento (DORSEY et al., 2016), como o Brasil.

Um estudo realizado em uma cidade brasileira apontou uma prevalência de 3,3% da DP entre pessoas idosas (BARBOSA et al. 2006). De acordo com o Instituto Brasileiro de Geografia e Estatística (IBGE), há, aproximadamente, 30 milhões de idosos no Brasil (CONTINUA, 2020). Se a prevalência encontrada nesse estudo for extrapolada para a população de idosos do Brasil, projeta-se que aproximadamente um milhão de brasileiros tenham DP. Além disso, as estimativas indicam que a incidência da DP vai, aproximadamente, dobrar até 2030 (DORSEY et al., 2007).[

A DP é uma doença degenerativa e progressiva do sistema nervoso central que causa incapacidades motoras e não motoras (KALIA et al., 2015). A neuropatologia da DP é multifatorial e heterogênea, mas há alguns marcos patológicos da doença (MCGREGOR et al., 2019). Um marco neuropatológico da DP é a presença de inclusões intracelulares, chamadas corpos de Lewy, decorrente da agregação da proteína α-

synuclein (KALIA et al., 2015). O local mais conhecido de deposição dos corpos de Lewy e neurodegeneração é o mesencéfalo, especificamente os neurônios dopaminérgicos da substância negra parte compacta e, em menor intensidade, a área tegmentar ventral (MCGREGOR et al., 2019). Com a perda de neurônios dopaminérgicos na parte compacta da substância negra ocorre a redução da dopamina no sistema nigroestriatal (KALIA et al., 2015). Por fim, outros sistemas não dopaminérgicos também são afetados com a progressão da doença, como projeções colinérgicas, serotoninérgicas e noradrenérgicas (MCGREGOR et al., 2019; SCHAPIRA et al., 2017).

A DP apresenta quatro sinais motores clássicos, também conhecidos como sinais cardinais. Estes sinais motores clássicos são a bradicinesia, rigidez, instabilidade postural e tremor de repouso (KALIA et al., 2015; MORRIS et al., 2000; SCHEKMAN et al., 2001; SHULMAN et al., 2011). Além disso, devido a essa característica multifatorial e heterogênea, a DP também afeta outros sistemas, e consequentemente, ocasiona várias deficiências não motoras, como alterações ventilatórias e respiratórias, déficits cognitivos, fadiga, disfunção autonômica, distúrbios do sono, alterações gastrointestinais, dentre outras (KALIA et al., 2015).

A Classificação Internacional de Funcionalidade, Incapacidade e Saúde (CIF) (ORGANIZAÇÃO MUNDIAL DE SAÚDE, 2003) fornece uma importante estrutura tanto para descrever a funcionalidade, quanto para orientar o estabelecimento de metas terapêuticas e planos de intervenção, sendo mundialmente utilizada em indivíduos com DP. Em um estudo conduzido por Raggi et al. (2011), indivíduos com DP reportaram deficiências em estruturas e funções corporais, como nas funções da potência e do tônus muscular, e limitações de atividade e restrições na participação social, como limitações na caminhada e restrição para utilizar transportes (RAGGI et al. 2011). Além disso,esses indivíduos identificaram a presença de fatores ambientais que foram qualificados como

barreiras ou facilitadores, como atitudes individuais de membros da família e sistemas e políticas de serviços de seguridade social (RAGGI et al. 2011).

1.2. Distúrbios Respiratórios na Doença de Parkinson

Distúrbios respiratórios e ventilatórios são sinais e sintomas não motores comumente observados em indivíduos com DP. Segundo estudos, pessoas com DP comumente apresentam fraqueza muscular respiratória (HAAS et al., 2004; GUEDES et al., 2012; PAL et al., 2007; SANTOS et al. 2019), função pulmonar reduzida, alteração da cinemática da caixa torácica, sensibilidade laríngea reduzida e reflexo de tosse comprometido (EBIHARA et al., 2003; FONTANA et al., 1998; PITTS et al., 2008). A prevalência de distúrbios respiratórios na DP ainda é subestimada. Em uma revisão da literatura, foi relatado que a prevalência de distúrbios restritivos em indivíduos com DP variou de 28% a 94%, e os distúrbios obstrutivos variaram de 6,7% a 67% (D'ARRIGO et al., 2020).

A fisiopatologia dos distúrbios respiratórios na DP ainda é incerta. As características biomecânicas da DP podem estar relacionadas aos disfunções respiratórios encontrados nesses indivíduos, como alterações posturais e rigidez na musculatura torácica, redução da mobilidade torácica (CARDOSO et al., 2002), alterações na ativação e coordenação dos músculos das vias aéreas superiores (MONTEIRO et al., 2014). Além disso, tremores (BROWN et al., 1997) e bradicinesia podem comprometer a função respiratória em indivíduos com DP (CARDOSO et al., 2002; GUEDES et al., 2012).

Alterações neuropatológicas também têm sido associadas a esses distúrbios respiratórios. Conforme descrito anteriormente, uma das características neuropatológicas da DP é a depleção de neurônios dopaminérgicos localizados na substância negra do mesencéfalo. Essa destruição neuronal ocorre também no encéfalo, como nos núcleos do

tronco cerebral que controlam o sono e a respiração. Além disso, agregação da proteína α-synuclein ocorre primeiramente no bulbo, e posteriormente, nos centros respiratórios (BRAAK H., 2003; DOCU AXELERAD et al., 2021).

Tem sido amplamente descrito na literatura científica que indivíduos com DP apresentam redução da força muscular respiratória quando comparados a indivíduos saudáveis do mesmo sexo e idade. Guedes e cols. (2012) encontraram valores de pressão inspiratória máxima (PImáx) e pressão expiratória máxima (PEmáx) significativamente menores em indivíduos com DP (PImáx (homens)= 65,0±17,9cmH_2O; PImáx (mulheres)= 55,6±18,8cmH_2O; PEmáx (homens)= 81,9±14,7cmH_2O; PEmáx mulheres= 64,4±21,9cmH_2O) quando comparados a indivíduos saudáveis do mesmo sexo e idade (PImáx (homens)= 86,9±6,2cmH_2O; PImáx (mulheres)= 70,0±14,1cmH_2O; PEmáx (homens)= 120,0 ±29,4cmH_2O; PEmáx (mulheres)= 105,6±19,4cmH_2O) (GUEDES et al. 2012).

Estudos já demonstraram que a redução da força muscular respiratória está presente nas fases iniciais da DP (BAILLE et al., 2019) e que se intensifica com a progressão da doença (SANTOS et al. 2019). Embora a disfunção respiratória seja frequente e potencialmente grave, ela pode não ser observada precocemente devido ao reduzido nível de atividade física dos indivíduos com DP (HAAS et al., 2004; HOVESTADT et al., 1989; SATHYAPRABHA et al., 2005). Com o declínio no nível de atividade física, esses indivíduos podem não realizar atividades que demandam um gasto energético suficiente para provocar adaptações respiratórias que promovam manifestações da disfunção respiratória (VAN NIMWEGEN et al., 2011). Com isso as abordagens voltadas para melhora da força muscular respiratória comumente são iniciadas em fases mais avançadas de acometimento..

Conforme descrito anteriormente, indivíduos com DP podem apresentar força

muscular reduzida tanto no grupo inspiratório quanto no expiratório. Ambos os grupos musculares são essenciais para manter a função respiratória adequada. Em indivíduos com DP já foi observado que a fraqueza muscular inspiratória predispõe ao aparecimento de atelectasias e retenção de secreção, sendo este um importante fator de risco para infecções (SAPIENZA et al., 2003). Na fraqueza muscular expiratória, há risco aumentado de acúmulo de secreções e desenvolvimento de complicações respiratórias, como pneumonia (BACKSTROM et al., 2018). Portanto, tanto a força muscular inspiratória quanto a expiratória devem ser medidas em indivíduos com DP e, quando for observada fraqueza muscular, intervenções para aumentar a força muscular de ambos os grupos musculares devem ser realizadas.

1.3. Treinamento Muscular Respiratório

Várias intervenções terapêuticas têm sido utilizadas para aumentar a força muscular respiratória em indivíduos com DP (HAAS et al. 2004; KILLIAN et al., 1984; MCMAHON et al. 2020; SANTOS et al. 2011). Uma revisão sistemática com metanálise avaliou os efeitos de intervenções não farmacológicas na função respiratória nessa população (MCMAHON et al. 2020). Nessa revisão sistemática foram incluídos estudos que avaliaram os efeitos de diversos tipos de intervenções, como exercícios aeróbicos, ioga, ciclismo, fortalecimento muscular periférico e fortalecimento específico da musculatura respiratória (treinamento da musculatura inspiratória e expiratória) (MCMAHON e outros 2020).

Os resultados dessa meta-análise demonstraram que os exercícios globais e o treinamento muscular respiratório tiveram um aumento estatisticamente significativo na força muscular respiratória, pico de fluxo expiratório e resistência muscular respiratória (MCMAHON et al. 2020). Além disso, o treinamento muscular respiratório aumentou significativamente o pico de fluxo da tosse e a dispneia, mas não foi possível realizar uma

metanálise para esses desfechos devido à heterogeneidade dos dados (MCMAHON et al. 2020).

O treinamento muscular respiratório tem o potencial de melhorar a função respiratória (MCMAHON et al. 2020). Revisões sistemáticas com meta-análise já demonstraram que o treinamento muscular respiratório é eficaz em aumentar a função respiratória de indivíduos com diferentes condições de saúde, como indivíduos com doença pulmonar obstrutiva crônica (SHAFFER et al., 1995), insuficiência cardíaca (MCELFRESH et al ., 2012), acidente vascular cerebral (MENEZES et al., 2016) e na ventilação mecânica (ELKINS et al., 2015). Em indivíduos com DP, uma revisão sistemática de ensaios clínicos aleatorizados (ECA) investigou os efeitos do fortalecimento muscular respiratório na função respiratória (RODRÍGUEZ et al. 2020). Apenas cinco estudos provenientes de três ECAs diferentes foram incluídos. Desses cinco estudos, um investigou o efeito do treinamento muscular inspiratório na força muscular inspiratória, resistência muscular respiratória, redução da dispneia e qualidade de vida (INZELBERG et al. 2005). Dois estudos provenientes de um único ECA avaliaram o efeito do fortalecimento muscular expiratório na deglutição (TROCHE et al., 2010), força muscular expiratória e função pulmonar (SAPIENZA et al., 2011). Além disso, em outros dois estudos provenientes de um único ECA, foi investigada a eficácia do treinamento muscular respiratório no pico de fluxo de tosse, força muscular inspiratória e expiratória (REYES et al., 2018) e parâmetros fonatórios (REYES et al., 2019).

Nesses estudos anteriores, a carga de treinamento variou de 15% a 60% da PImáx (INZELBERG et al. 2005) e de 50% a 75% da PEmáx (REYES et al. 2018; REYES et al. 2019; SAPIENZA et al . 2011; TROCHE et al. 2010), a duração variou de quatro (SAPIENZA et al. 2011; TROCHE et al. 2010) a 12 semanas (INZELBERG et al. 2005) e a frequência de cinco a seis vezes por semana (INZELBERG et al . 2005; REYES et al.

2018; REYES et al. 2019). Não foi possível avaliar a superioridade de um protocolo sobre o outro devido à heterogeneidade nas medidas de resultado. A revisão sistemática demonstrou que o treinamento muscular respiratório é eficaz na melhora da força e resistência muscular respiratória, função pulmonar, deglutição, dispneia e parâmetros fonatórios de indivíduos com DP (RODRÍGUEZ et al., 2020). No entanto, um pequeno número de estudos foi encontrado (apenas três ECAs). Além disso, foi demonstrado que os estudos existentes têm qualidade metodológica moderada e heterogeneidade em suas medidas de desfecho (RODRÍGUEZ et al., 2020). Portanto, os autores concluíram que as evidências são limitadas para qualquer recomendação para a prática clínica, sugerindo a realização de outros ECAs (RODRÍGUEZ et al., 2020).

Nesses estudos, foi encontrado aumento da PImáx, variando de -1,28cmH_2O (INZELBERG et al., 2005) a -16cmH_2O (REYES et al., 2018). Além disso, foi observado um aumento na PEmáx de 15,5cmH_2O (REYES et al., 2018) para 27,97cmH_2O (SAPIENZA et al., 2011) quando o treinamento muscular expiratório foi realizado. Em outros estudos que investigaram o efeito do treinamento muscular respiratório de alta intensidade em outras populações, foi encontrada melhora da PImáx de -43cmH_2O e da PEmáx de 51cmH_2O, ou seja, aproximadamente o dobro (GOMES NETO et al., 2018; HILL et al. , 2006; PARREIRAS DE MENEZES et al., 2019). Além disso, os benefícios do treinamento muscular respiratório sobre outros desfechos funcionais (percepção de dispneia, fadiga e capacidade de exercício) parecem ser obtidos quando um alto volume de treinamento (carga, duração e frequência) é aplicado (GOMES NETO et al., 2018; HILL et al., 2006). Portanto, os efeitos do treinamento muscular respiratório com maiores intensidades (carga, intensidade e volume) ainda precisam ser investigados em indivíduos com DP.

Não foram encontrados estudos que investigaram os efeitos do treinamento

muscular respiratório sobre a fadiga e a capacidade de exercício de indivíduos com DP. Em indivíduos com outras doenças neurológicas, o treinamento muscular respiratório demonstrou melhorar o desempenho nas atividades de vida diária (CHEN et al., 2016), capacidade de exercício (SUTBEYAZ et al., 2010) e qualidade de vida (SUTBEYAZ et al., 2010). , 2010). Em indivíduos com DP, a fraqueza muscular respiratória apresentou correlação estatisticamente significativa com a capacidade de exercício (HAAS et al., 2004). Portanto, o treinamento muscular respiratório pode melhorar esses importantes resultados nessa população.

Por fim, não foram encontrados estudos que investigassem a associação desses treinamentos (inspiratório mais expiratório) nessa população. A realização de treinamento muscular inspiratório e expiratório utilizando um único equipamento é mais barata e viável, leva menos tempo e é de mais fácil compreenção. Assim, é importante investigar os efeitos do treinamento muscular respiratório de alta intensidade (associação do treinamento muscular inspiratório e expiratório) na força muscular inspiratória e expiratória, resistência muscular inspiratória, pico de fluxo de tosse, dispneia, fadiga, capacidade de exercício e qualidade de respiração. vida nesta população**.**

2. OBJETIVOS

• Objetivo primário: Investigar os efeitos do treinamento muscular respiratório de alta intensidade (associação do treinamento muscular inspiratório e expiratório) na força muscular inspiratória e expiratória de indivíduos com DP.

• Objetivo secundário: Investigar os efeitos do treinamento muscular respiratório de alta intensidade (associação do treinamento muscular inspiratório e expiratório) na resistência muscular inspiratória, pico de fluxo de tosse, dispneia, fadiga, capacidade de exercício e qualidade de vida nessa população.

3.0. MÉTODOS

Um estudo prospectivo, randomizado, controlado, com alocação oculta, proporção de alocação 1:1, e análise de intenção de tratar, será realizado no Departamento de Fisioterapia da Universidade Federal de Minas Gerais (UFMG).

O projeto será submetido ao Comitê de Ética em Pesquisa da UFMG. Esse ensaio será registrado em www.ClinicalTrials.gov e realizado de acordo com os *Consolidated Standards of Reporting Trials* (CONSORT) (SCHULZ et al. 2010). Todos os indivíduos serão instruídos sobre os procedimentos que serão realizados e assinarão um termo de consentimento livre e esclarecido. Durante o estudo, serão observadas as medidas de prevenção à disseminação do coronavírus, como atendimento de apenas um participante por vez, uso de máscara e álcool gel (COMITÊ PERMANENTE DE ENFRENTAMENTO DO NOVO CORONAVÍRUS DA UFMG, 2020; MINISTÉRIO DA SAÚDE, 2020).

3.1. Participantes

Uma amostra de conveniência será recrutada na comunidade geral da cidade de Belo Horizonte a partir de contato com ambulatórios, clínicas, hospitais, centros de saúde, associações, grupos de pesquisa e projetos de extensão. Serão incluídos indivíduos segundo os seguintes critérios: DP idiopática, diagnosticada por um neurologista; com capacidade de deambular de forma independente com ou sem dispositivos auxiliares; idade> 50 anos; em uso de medicação anti- parkinsoniana e que estejam medicamente estáveis a pelo menos 6 meses; classificados entre estágios 1 a 3 da escala de Hoehn e Yahr modificada (SCHENKMAN et al., 2001); que apresentem pressão inspiratória máxima <80 cmH_2O ou pressão expiratória máxima <90 cmH_2O (FARRERO et al., 2013). Serão excluídos indivíduos que estejam realizando

treinamento muscular respiratório ou o tenham realizado nas últimas quatro semanas; possíveis alterações cognitivas avaliadas pelo Mini-Exame do Estado Mental; indivíduos em uso de estimulação cerebral profunda (DBS); fumantes ou que pararam de fumar há menos de seis meses; tenham sido acometidos por infecções respiratórias ou cardíacas no último mês; e indivíduos que apresentem outros distúrbios neurológicos, musculoesqueléticos e respiratórios que interfiram na execução dos testes e intervenções propostas.

3.2. Randomização

Os participantes serão alocados em dois grupos (experimental e controle). A sequência de alocação será gerada por um site (www.randomization.com). Um assistente de pesquisa não envolvido no recrutamento fará a randomização e colocará a sequência em envelopes opacos, numerados e selados. Um examinador treinado, cego para a sequência de alocação do grupo, coletará as medidas pré-intervenção. Posteriormente, um assistente de pesquisa treinado revelará o conteúdo desses envelopes.

Algumas medidas serão tomadas na tentativa de cegamento dos voluntários quanto à intervenção recebida/alocação do grupo. Na avaliação inicial, os indivíduos não serão informados sobre a diferença entre as intervenções. Além disso, para não revelar detalhes da intervenção para os grupos, todos os dispositivos serão envolvidos com um material opaco, para que a carga de treinamento respiratório não seja visualizada. Por fim, na avaliação de um mês após o final da intervenção (semana 12) os indivíduos serão questionados se eles desconfiavam em qual grupo foram alocados. O mesmo procedimento será realizado com os avaliadores. A taxa de acertos será computada, e utilizada como uma medida indireta de sucesso do cegamento.

3.3. Intervenção

Os participantes serão submetidos a treinamento muscular inspiratório e expiratório de alta intensidade (grupo experimental), ou uma intervenção placebo. Ambos os grupos farão o mesmo protocolo de exercícios, a única diferença entre os dois grupos será a presença/ausência da carga imposta durante o treinamento. Será utilizado o aparelho Orygen-Dual Valve® (Forumed S.L., Girona, Espanha), que proporciona cargas de treinamento de até 70 cmH_2O, uma vedação adequada, através de um bocal confortável e flexível.

Os indivíduos realizarão uma intervenção domiciliar, dividida em duas sessões diárias de 20 minutos (manhã e tarde), totalizando 40 minutos por dia, sete vezes por semana, durante oito semanas. Cada sessão diária será composta por quatro blocos de três minutos, com dois minutos de descanso entre os blocos.

Para monitorar a adesão ao protocolo, os indivíduos receberão um diário de treinamento, no qual serão registradas a duração e a percepção subjetiva do esforço por meio da escala de Borg, para cada dia de intervenção. Se necessário, um cuidador será instruído a auxiliar o indivíduo no preenchimento do diário.

3.3.1. Grupo Experimental

O treinamento será realizado usando o Orygen Dual Valve® (Forumed S.L., Girona, Espanha). Os indivíduos realizarão uma intervenção domiciliar, dividida em duas sessões diárias de 20 minutos (manhã e tarde), totalizando 40 minutos por dia, sete vezes por semana, durante oito semanas. Cada sessão diária será composta por quatro blocos de três minutos, com dois minutos de descanso entre os blocos. A carga de treinamento inicial será 60% das pressões respiratórias máximas (60% da PImáx e PEmáx). O escore

de Borg também será considerado para ajustar a intensidade do treinamento, com valores alvos de 4 a 6. Uma vez por semana, um pesquisador treinado visitará suas residências, medirá PImáx e PEmáx e progredirá a carga de treinamento para garantir que 60% dos novos valores pressóricos sejam mantidos.

3.3.2. Grupo Controle

O grupo controle também realizará os exercícios com o aparelho Orygen Dual Valve® (Forumed S.L., Girona, Espanha). Será implementada uma intervenção placebo: a resistência inicial do aparelho será de 0cmH_2O, que será mantida durante todo o período de intervenção, portanto não haverá progressão de carga. Todos os procedimentos adotados com o grupo experimental, inclusive a visita domiciliar semanal, também serão realizados com os indivíduos do grupo controle. Entretanto, não haverá mudança real na carga de treinamento. Todos os aparelhos serão envoltos em material opaco para que não seja visualizada a carga ou possível carga de treinamento respiratório.

3.4. Mensuração dos Desfechos

As mensurações serão realizadas na avaliação inicial (semana 0), imediatamente após o treinamento (semana 8) e um mês após o treinamento (semana 12). Na avaliação inicial serão coletados dados clínicos e demográficos de todos os indivíduos, identificação e caracterização da amostra.

Nas três avaliações serão realizadas medidas de PImáx e PEmáx, resistência muscular inspiratória, pico de fluxo de tosse, dispnéia, fadiga, capacidade de exercício e qualidade de vida. Todas essas medidas serão realizadas por um examinador cegado para a alocação dos grupos. Todos os participantes serão instruídos a não comentar informações sobre o treinamento recebido.

3.5. Instrumentos

3.5.1. Desfechos Primários

3.5.1.1. Força muscular inspiratória

A força muscular inspiratória será mensurada por meio da PImáx. As medidas serão realizadas com manovacuômetro digital (NEPEB-LabCare/UFMG, Brasil) e reportadas em cmH_2O (PESSOA et al., 2014; PESSOA et al., 2015). A determinação das variáveis do manovacuômetro é realizada por meio do software MANOVAC 4.0. O manovacuômetro é o instrumento padrão ouro para mensurar a força muscular respiratória (PESSOA et al., 2014). Para registrar as pressões inspiratórias, os indivíduos irão realizar inspirações contra uma via aérea obstruída dentro do bucal. A coleta será realizada com o indivíduo sentado em uma cadeira, com os pés e costas apoiados e o tronco em um ângulo de 90 graus em relação ao quadril. Uma boquilha convencional e clipe nasal serão utilizados (PESSOA et al., 2015). Os indivíduos serão instruídos a respirar confortavelmente duas a três vezes, a nível de volume corrente. Duas medidas de familiarização serão realizadas, e em seguida, serão realizadas cinco medidas aceitáveis, com duração de pelo menos um segundo. A maior pressão de três medidas reprodutíveis, com menos de 10% de variabilidade, será registrada e utilizada nas análises (PESSOA et al., 2015).

3.5.1.2. Força muscular expiratória

A força muscular expiratória será mensurada por meio da PEmáx. A PEmáx será medida com o mesmo manovacuômetro digital (NEPEB-LabCare/UFMG, Brasil), e reportada em cmH_2O (PESSOA et al., 2014; PESSOA et al., 2015). Para registrar as pressões expiratórias, os indivíduos realizarão expirações contra uma via aérea obstruída dentro do bocal (PESSOA et al., 2015). O protocolo de coleta será o mesmo descrito

anteriormente para avaliação da força muscular inspiratória. A maior pressão de três medidas reprodutíveis, com menos de 10% de variabilidade, será registrada e utilizada na análise dos dados (PESSOA et al., 2015).

3.5.2. Desfechos Secundários

3.5.2.1. Resistência muscular respiratória

A resistência muscular inspiratória será medida pelo número de respirações que o indivíduo conseguir realizar. Os indivíduos serão instruídos a respirar contra uma carga inspiratória submáxima (50% da PImáx), até ocorrer a fadiga ou até o limite de sete minutos (CHARUSUSIN et al., 2013). Será utilizado o instrumento POWERbreathe® KH1, e serão seguidas recomendações para o uso do equipamento (CHARUSUSIN et al., 2013). O número total de repetições que o indivíduo conseguiu realizar será utilizado para análises (CHARUSUSIN et al., 2013).

3.5.2.2. Pico de fluxo da tosse

As medidas de pico de fluxo da tosse serão realizadas com o medidor de pico de fluxo expiratório máximo (Mini-Wright Peak Expiratory Flow Meter) (FREITAS et al., 2010). Para avaliação dessa medida, o indivíduo deverealizar uma inspiração profunda, ou seja, na capacidade pulmonar total, e em seguida, realizar uma tosse o mais vigorosa possível (BACH et al., 2006). Serão realizadas pelo menos três medições que devem ter um máximo diferente de 5% entre elas (FREITAS et al., 2010; PEREIRA et a., 2002). O valor mais alto, em L/min, será registrado e utilizado nas análises (FREITAS et al., 2010; PEREIRA et al., 2002).

3.5.2.3. Dispneia

A dispneia será avaliada utilizando o instrumento Medical Research Council (MRC) (KOVELIS et al., 2008). A MRC é um instrumento em que o indivíduo relata o seu grau subjetivo de dispneia. Os valores variam de 1 a 5, sendo que em 1 o indivíduo só sofre de falta de ar durante exercícios intensos e em 5 sente tanta falta de ar que não sai mais de casa, ou sente falta de ar quando está se vestindo (KOVELIS et al., 2008). Este instrumento já foi traduzido e adaptado para o português e apresenta adequada validade para população brasileira (KOVELIS et al., 2008). A pontuação total será utilizada nas análises.

3.5.2.4. Fadiga

Para avaliar a fadiga será utilizado o instrumento Fatigue Severity Scale (FSS). Este instrumento mensura o impacto da fadiga nas atividades de vida diária. Na FSS são apresentadas nove afirmações e o indivíduo deve pontuar o quanto ele concorda com a afirmação. A pontuação varia de 1 a 7, onde 1 ele discorda completamente e 7 ele concorda plenamente. A FSS é recomendada para avaliação da fadiga em indivíduos com DP (FRIEDMAN et al., 2010) e sua versão adaptada transculturalmente para o português apresenta adequadas propriedades de medida para mensuração da fadiga nessa população (FRIEDMAN et al., 2010; VALDERRAMAS et al., 2012). A pontuação será utilizada nas análises.

3.5.2.5. Capacidade de Exercício

A capacidade de exercício será avaliada utilizando o teste de caminhada de seis minutos (TC6), que apresenta adequadas propriedades de medida para avaliação deste desfecho em indivíduos com DP (BLOEM et al., 2016; STEFFEN et al., 2008). O TC6 será realizado em um corredor de 30 metros, delimitado por dois cones. Os indivíduos

serão orientados a caminhar a maior distância possível em seis minutos, e a cada minuto de teste serão fornecidos estímulos padronizados. Serão realizados dois testes com intervalo de 30 minutos entre eles, de acordo com as diretrizes da European Respiratory Society/ American Thoracic Society (HOLLAND et al., 2014). A distância total percorrida, em metros, será utilizada nas análises.

3.5.2.6. Qualidade de vida

O *Parkinson's Disease Questionnaire-39* (PDQ-39) é um instrumento específico para avaliação da qualidade de vida na DP (JENKINSON et al., 1997). Este instrumento é composto por 39 itens, e é dividido em oito dimensões. Cada item apresenta cinco opções de respostas, e os escores em cada item variam de 0 (nunca) a 4 (sempre ou é impossível para mim) (JENKINSON et al., 1997). A pontuação para cada domínio varia de 0 (zero) a 100 (cem), onde o zero, significa melhor e cem uma pior qualidade de vida (JENKINSON et al., 1997). A versão traduzida para o português e adaptada transculturalmente para a população brasileira apresenta adequada validade de constructo e confiabilidade inter-examinadores (LANA et al., 2007). A pontuação total será utilizada para as análises.

3.6. Calculo amostral

O cálculo do amostral foi realizado considerando as medidas de desfecho primário (força muscular inspiratória e expiratória). O tamanho do efeito para o treinamento muscular inspiratório foi derivado de um ECA com população e intervenção semelhantes. Considerando um nível de significância (α) de 5% e um *power* de 0,80, são necessários treze participantes por grupo (26 participantes). O tamanho do efeito para o treinamento muscular expiratório também foi derivado de um ECA com população e

intervenção semelhantes. Considerando um nível de significância (α) de 5% e um *power* de 0,80, são necessários quatorze participantes por grupo (28 participantes). Assim, foi definido um tamanho amostral de 28 indivíduos (14 em cada grupo) (o maior tamanho amostral calculado). Assumindo-se uma perda amostral de 20% indivíduos, estabeleceu-se uma amostra total de 34 pessoas no total (17 em cada grupo).

3.7. Análise estatística

Todas as análises estatísticas serão realizadas por um examinador independente, cegado quanto a todos os procedimentos realizados no estudo, inclusive com relação à alocação dos grupos. Todas as análises serão realizadas com intenção de tratar. Estatísticas descritivas serão utilizadas para caracterização da amostra. A normalidade da distribuição dos dados será testada para todas as variáveis numéricas contínuas. O test-t de students será empregado para investigar diferenças entre os grupos na linha de base em relação às características clínicas e demográficas. ANOVA two-way com medidas repetidas será utilizada para avaliar a diferenças entre grupos nos três momentos de avaliação (semana 0, semana 8 e semana 12) para as variáveis força muscular inspiratória e expiratória, endurance muscular respiratório, pico de fluxo da tosse, nível de atividade física, parâmetros do sono e capacidade de exercício. O teste Mann-Whitney U será utilizado para avaliar a diferença entre grupos para as variáveis: fadiga, dispneia e qualidade de vida. Todas as análises serão realizadas com o programa estatístico SPSS 20.0 e será considerado um α=0,05.

REFERÊNCIAS

BACH, J. R. et al. Expiratory flow maneuvers in patients with neuromuscular diseases. **American journal of physical medicine & rehabilitation**, v. 85, n. 2, p. 105-111, 2006.

BÄCKSTRÖM, D. et al. Early predictors of mortality in parkinsonism and Parkinson disease: A population-based study. **Neurology,** v. 91, n. 22, p. e2045-e2056, 2018.

BAILLE, G. et al. Dyspnea: An underestimated symptom in Parkinson's disease. **Parkinsonism Relat Disord,** v. 60, p. 162-166, 2019.

BARBOSA, M. T. et al. Parkinsonism and Parkinson's disease in the elderly: A community-based survey in Brazil (the Bambuí study). **Movement Disorders,** v. 21, n. 6, p. 800–808, 2006.

BLOEM, B. R. et al. Measurement instruments to assess posture, gait, and balance in Parkinson's disease: Critique and recommendations. **Mov Disord,** v. 31, n. 9, p. 1342-55, 2016.

BORG, G. A. Psychophysical bases of perceived exertion. **Med Sci Sports Exerc,** v. 14, n. 5, p. 377-81, 1982.

BRAAK, H. et al. Staging of brain pathology related to sporadic Parkinson's disease. **Neurobiol Aging,** v. 24, n. 2, p. 197-211, 2003.

BROWN, P.; CORCOS, D. M.; ROTHWELL, J. C. Does parkinsonian action tremor contribute to muscle weakness in Parkinson's disease? **Brain,** v. 120, p. 401-8, 1997.

CARDOSO, S. R.; PEREIRA, J. S. Analysis of breathing function in Parkinson's disease. **Arq Neuropsiquiatr,** v. 60, n. 1, p. 91-5, 2002.

CHARUSUSIN, N. et al. Inspiratory muscle training protocol for patients with chronic obstructive pulmonary disease (IMTCO study): a multicentre randomised controlled trial. **BMJ Open,** v. 3, n. 8, 2013.

CHEN, P.C. et al. Inspiratory muscle training in stroke patients with congestive heart failure: A CONSORT-compliant prospective randomized single-blind controlled trial. **Medicine (Baltimore)**, v. 95, n. 37, p. e4856, 2016.

COMITÊ PERMANENTE DE ENFRENTAMENTO DO NOVO CORONAVÍRUS DA UFMG, 2020. Protocolo de biossegurança e adequação do espaço físico na UFMG. 2020.

CONTINUA, IBGE PNAD. Características gerais dos domicílios e dos moradores 2019. 2020.

D'ARRIGO, A. et al. Respiratory dysfunction in Parkinson's disease: a narrative review. **ERJ Open Research,** v. 6, n. 4, 2020.

DOCU AXELERAD, A. et al. Respiratory Dysfunctions in Parkinson's Disease Patients. **Brain Sciences,** v. 11, n. 5, p. 595, 2021.

DORSEY, E. R. et al. Projected number of people with Parkinson disease in the most populous nations, 2005 through 2030. **Neurology,** v. 68, n. 5, p. 384-6, 2007.

DORSEY, E.R. et al. Global, regional, and national burden of Parkinson's disease, 1990–2016: a systematic analysis for the Global Burden of Disease Study 2016. **The Lancet Neurology,** v. 17, n. 11, p. 939-953, 2018.

EBIHARA, S. et al. Impaired efficacy of cough in patients with Parkinson disease. **Chest,**

v.124, n. 3, p. 1009-15, 2003.

ELKINS M.; DENTICE R. Inspiratory muscle training facilitates weaning from mechanical ventilation among patients in the intensive care unit: a systematic review. **J Physiother,** v. 61, n. 3, p. 125-134, 2015.

FONTANA, G. A. et al. Defective motor control of coughing in Parkinson's disease. **Am J Respir Crit Care Med,** v. 158, n. 2, p. 458-64, 1998.

FREITAS, et al. Relationship between cough strength and functional level in elderly. **Brazilian Journal of Physical Therapy**, v. 14, p. 470-476, 2010.

FRIEDMAN, J. H. et al. Fatigue rating scales critique and recommendations by the Movement Disorders Society task force on rating scales for Parkinson's disease. **Mov Disord,** v. 25, n. 7, p. 805-22, 2010.

GUEDES, L. U. et al. Respiratory changes in Parkinson's disease may be unrelated to dopaminergic dysfunction. **Arq Neuropsiquiatr,** v. 70, n. 11, p. 847-51, 2012.

GROUP, G. N. D. C. Global, regional, and national burden of neurological disorders during 1990-2015: a systematic analysis for the Global Burden of Disease Study 2015. **Lancet Neurol,** v. 16, n. 11, p. 877-897, 2017.

HAAS, B. M. et al. Effects of respiratory muscle weakness on daily living function, quality of life, activity levels, and exercise capacity in mild to moderate Parkinson's disease. **Am J Phys Med Rehabil,** v. 83, n. 8, p. 601-7, 2004.

HOLLAND, A. E. et al. An official European Respiratory Society/American Thoracic Society technical standard: field walking tests in chronic respiratory disease. **Eur Respir**

J, v. 44, n. 6, p. 1428-46, 2014.

HOVESTADT, A. et al. Pulmonary function in Parkinson's disease. **Journal of Neurology, Neurosurgery & Psychiatry,** v. 52, n. 3, p. 329-333, 1989.

INZELBERG, R. et al. Inspiratory muscle training and the perception of dyspnea in Parkinson's disease. **Canadian journal of neurological sciences,** v. 32, n. 2, p. 213-217, 2005.

JENKINSON, C. et al. The Parkinson's Disease Questionnaire (PDQ-39): development and validation of a Parkinson's disease summary index score. **Age and ageing**, v. 26, n. 5, p. 353-357, 1997.

KALIA, L.; LANG, A. Parkinson’s disease. **Lancet [Internet].** 2015; 386 (9996): 896–912.

KILLIAN, K. J. et al. Effect of increased lung volume on perception of breathlessness, effort, and tension. **J Appl Physiol Respir Environ Exerc Physiol,** v. 57, n. 3, p. 686-91, 1984.

KOVELIS, D. et al. Validation of the Modified Pulmonary Functional Status and Dyspnea Questionnaire and the Medical Research Council scale for use in Brazilian patients with chronic obstructive pulmonary disease. **J Bras Pneumol,**v. 34, n. 12, p. 1008-18, 2008.

LANA, R. C. et al. Percepção da qualidade de vida de indivíduos com doença de Parkinson através do PDQ-39. **Brazilian Journal of Physical Therapy**, v. 11, p. 397-402, 2007.

MCELFRESH, J. et al. Inspiratory muscle training in patients with heart failure: a systematic review. **Cardiopulm Phys Ther J.** v. 23, n. 3, p. 29-36, 2012.

MCGREGOR, M. M.; NELSON, A. B. Circuit mechanisms of Parkinson’s disease. **Neuron,** v. 101, n. 6, p. 1042-1056, 2019.

MCMAHON, et al. Nonpharmacological Interventions for Respiratory Health in Parkinson’s Disease: A Systematic Review and Meta-analysis. **European Journal of Neurology,** 2020.

MENEZES, et al. High-intensity respiratory muscle training improves strength and dyspnea post stroke: a double-blind randomized trial. **Archives of physical medicine and rehabilitation**, v. 100, n. 2, p. 205-212, 2019.

MENEZES, et al. Respiratory muscle training increases strength of respiratory muscles and reduces the occurrence of respiratory complications after stroke: a systematic review. **J Physiother,** v. 62, n. 3, p. 138 -144, 2016.

MINISTÉRIO DA SAÚDE. PORTARIA Nº 1.565, DE 18 DE JUNHO DE 2020. Diário Oficial da União. Ed. 15, sessão 1, p. 64, 2020.

MONTEIRO, L. et al. Swallowing impairment and pulmonary dysfunction in Parkinson's disease: the silent threats. **J Neurol Sci,** v. 339, n. 1-2, p. 149-52, 2014.

MORRIS, M. E. Movement disorders in people with Parkinson disease: a model for physical therapy. **Phys Ther,** v. 80, n. 6, p. 578-97, 2000.

ORGANIZAÇÃO MUNDIAL DE SAÚDE – OMS; ORGANIZAÇÃO PANAMERICANA DE SAÚDE - OPAS. CIF - Classificação Internacional de

Funcionalidade, Incapacidade e Saúde. **São Paulo: Editora da Universidade de São Paulo**, 2003.

PAL, P. K. et al. Pattern of subclinical pulmonary dysfunctions in Parkinson's disease and the effect of levodopa. **Mov Disord,** v. 22, n. 3, p. 420-4, 2007.

PEREIRA, C. A. C. et al. SBPT. Diretrizes para testes de função pulmonar. **J Pneumol**, v. 29, n. 3, p. 207-221, 2002.

PESSOA, I. M. B. S. et al. Test-retest reliability and concurrent validity of a digital manovacuometer. **Fisioterapia e Pesquisa,** v. 21, n. 3, p. 236-242, 2014.

PESSOA, I. M. B. S. et al. Comparison of three protocols for measuring the maximal respiratory pressures. **Fisioterapia em Movimento,** v. 28, n. 1, p. 31- 39, 2015.

PITTS, T. et al. Voluntary cough production and swallow dysfunction in Parkinson's disease. **Dysphagia,** v. 23, n. 3, p. 297-301, 2008.

RAGGI, A. et al. Disability and profiles of functioning of patients with Parkinson's disease described with ICF classification. **International Journal of Rehabilitation Research,** v. 34, n. 2, p. 141-150, 2011.

REYES, A. et al. The effects of respiratory muscle training on peak cough flow in patients with Parkinson's disease: a randomized controlled study. **Clin Rehabil,** v. 32, n. 10, p. 1317- 1327, 2018.

REYES, A. et al. The Effects of Respiratory Muscle Training on Phonatory Measures in Individuals with Parkinson's Disease. **J Voice**, 2019.

RODRÍGUEZ, M. Á. et al. Should respiratory muscle training be part of the treatment of

Parkinson’s disease? A systematic review of randomized controlled trials. **Clinical Rehabilitation,** v. 34, n. 4, p. 429-437, 2020.

SANTOS, R. B. D. et al. Respiratory muscle strength and lung function in the stages of Parkinson's disease. **J Bras Pneumol,** v. 45, n. 6, p. e20180148, 2019.

SAPIENZA, C. et al. Respiratory strength training: concept and intervention outcomes. **Semin Speech Lang,** v. 32, n. 1, p. 21-30, 2011.

SATHYAPRABHA, T. N. et al. Pulmonary functions in Parkinson's disease. **Indian J Chest Dis Allied Sci,** v. 47, n. 4, p. 251-7, 2005.

SCHENKMAN, M. et al. Exercise for people in early- or mid-stage Parkinson disease: a 16-month randomized controlled trial. **Phys Ther**, v. 92, p. 1395- 1410, 2012.

SCHAPIRA, A. H. V. et al. Non-motor features of Parkinson disease. **Nature Reviews Neuroscience,** v. 18, n. 7, p. 435-450, 2017.

SCHULZ, K. F. et al. CONSORT 2010 statement: updated guidelines for reporting parallel group randomised trials. **Trials,** v. 11, n. 1, p. 1-8, 2010.

SHAFFER, T. H. et al. Respiratory muscle function, assessment, and training. **Physical therapy,** v. 61, n. 12, p. 1711-1723, 1981.

SHULMAN, J. M.; DE JAGER, P. L.; FEANY, M. B. Parkinson's disease: genetics and pathogenesis. **Annual Review of Pathology: Mechanisms of Disease,** v. 6, p. 193-222, 2011.

SUTBEYAZ, S.T. et al. Respiratory muscle training improves cardiopulmonary function and exercise tolerance in subjects with subacute stroke: a randomized controlled trial.

Clinical Rehabilitation, v. 24, n. 3, p. 240–250, 2010.

TROCHE, M. S. et al. Aspiration and swallowing in Parkinson disease and rehabilitation with EMST: a randomized trial. **Neurology,** v. 75, n. 21, p. 1912-9, 2010.

VALDERRAMAS, S. et al. Reliability and validity study of a Brazilian- Portuguese version of the fatigue severity scale in Parkinson's disease patients. **Arquivos de neuro-psiquiatria**, v. 70, p. 497-500, 2012.

VAN NIMWEGEN, M. et al. Physical inactivity in Parkinson's disease. **Journal of neurology**, v. 258, n. 12, p. 2214-2221, 2011.
