# Supplementary material for: Effects of high-intensity respiratory muscle training on respiratory muscle strength in individuals with Parkinson’s disease: Protocol of a randomized clinical trial
Source: PLoS One. 2023 Sep 8;18(9):e0291051. doi: 10.1371/journal.pone.0291051 (PMC10490961; doi:10.1371/journal.pone.0291051)
Supplement: Attachment — Submitted filename: Response to Reviewers.docx [file pone.0291051.s004.docx]

**RESPONSE TO THE editor**

**Ref:** PONE-D-23-05779

**Title:** “Effects of high-intensity respiratory muscle training on respiratory muscle strength in individuals with Parkinson's disease: protocol of a randomized clinical trial.”

**Journal Requirements:**

**When submitting your revision, we need you to address these additional requirements.**

**1. We note that your SPIRIT schedule has been uploaded as a figure other than Fig 1. In keeping with our submission guidelines, please upload your SPIRIT schedule as Fig 1 and amend your figure legends and captions as necessary. More information related to the relevant journal policy is available here:**

**<https://journals.plos.org/plosone/s/submission-guidelines#loc-guidelines-for-specific-study-types>**

We would like to thank for all comments and suggestions.

The SPIRIT schedule was loaded as Fig 1. The figure citation in text and the figure caption have been changed.

**2. We note that you did not include a copy of the original, (signed or stamped) dated IRB/research ethics committee approval letter or waiver for all study sites as an ‘Other’ file. In keeping with our submission guidelines, please upload the approval letter or waiver as an ‘Other’ file. More information related to the relevant journal policies is available from:** [**https://journals.plos.org/plosone/s/submission-guidelines#loc-guidelines-for-specific-study-types**](https://journals.plos.org/plosone/s/submission-guidelines%23loc-guidelines-for-specific-study-types)

We included a copy of the original (signed) dated research ethics committee approval letter as Other File (Other File - research ethics committee approval letter).

**3. We note you did not include a letter from an external funder confirming the funding award you received as an ‘Other’ file. In keeping with our submission guidelines, please upload the Funding letter you received as an ‘Other’ file. If you are unable to secure a letter from the funder confirming your funding award, please note this in your cover letter. More information related to the relevant journal policies is available from:** [**https://journals.plos.org/plosone/s/submission-guidelines#loc-guidelines-for-specific-study-types**](https://journals.plos.org/plosone/s/submission-guidelines%23loc-guidelines-for-specific-study-types)

All the financial support received was provided to pay to equipment, book and insume acquisition for the broader research project. For this reason, a letter from the funder confirming its granting of funding was not enclosed. This information was included in cover letter.
