# Supplementary material for: Effects of high-intensity respiratory muscle training on respiratory muscle strength in individuals with Parkinson’s disease: Protocol of a randomized clinical trial
Source: PLoS One. 2023 Sep 8;18(9):e0291051. doi: 10.1371/journal.pone.0291051 (PMC10490961; doi:10.1371/journal.pone.0291051)
Supplement: Attachment — Submitted filename: General comment.docx [file pone.0291051.s005.docx]

**General comment**

Thanks for the opportunity to review this manuscript. I have made some comments that I believe will help the authors to improve the protocol.

**Specific comments**

- In the introduction section. It is not clear by what mechanism reduced inspiratory/expiratory muscle strength and endurance may have an effect in reduced cough efficacy. In particular, the effect of respiratory muscle endurance on cough efficacy is not clear for the reader. Please clarify.
- I suggest to the authors that it is more accurate to state that in case of accumulation of mucus, an effective cough may reduce the risk of aspiration and a respiration infection.
- Dyspnoea in individuals with PD may have multiple causes. It is not clear that dyspnoea is related to inspiratory muscle weakness in individuals with PD. Dyspnoea is described in individuals with inspiratory muscle weakness and cardiometabolic reflex. This is not the case in PD. In addition, individuals with do not necessarily have inspiratory muscle weakness. I suggest being more accurate with the rationale of the study.
- Although previous studies have not reported or calculated effect sizes, as long as these studies provide the mean, standard deviation and sample size, it is possible to calculate the effect sizes. It is not clear what is the point that the authors try to make with this argument.
- In lines 104-105. This statement has no logical connection with the argument of the paragraph. Please note that a topic not yet investigated does not necessarily mean that the topic is relevant for research.
- In lines 125-126. It is not clear how the coordination of inspiratory and expiratory muscles will improve cough effectiveness. Please provide more details
- In the methods section, study design, what do the authors mean by superiority and parallel group trial? Please explain and check if those descriptors are relevant or add relevant information.
- What parameters make the intervention “high intensity”? Is there a number as a criterion for that classification?
- It is not clear why the authors used <80 and <90 cmH20 mip and mep for inclusion criteria. As it is, seems arbitrary.
- Regarding randomization to study groups. I suggest explaining if for example participants will be allocated using a simple randomisation method, the minimization method, bloke randomisation method, etcetera.
- The control group will be using the same training device as the intervention group? Is it possible to set this device at 0 cmH20? Please clarify.
- It is not clear why the authors intent to apply the same protocol to improve strength and endurance. It may be better to use a different protocol for each property.
- There may be no point in wrapping the device for the control group. Training without load will be evident for participants.
- It is certainly possible that not all individuals with PD may be able to start training at 60% of MIP. I suggest to the authors to include familiarisation weeks with the device.
- I suggest to the authors to use a different criterion to increase the training load. This is because the Borg scale may not be sensitive enough to detect the effort of participants.
- Is it important for the authors the number of efforts that participants can make in 20 minutes? It may be that in 20 minutes each participant makes different number of efforts. This may add some extra bias to the protocol. Please take this into consideration.
- I suggest to the authors to revise literature regarding test-retest, reliability and learning effect of mip and mep measurements. There is extensive literature indicating that a single measure of mip and mep underestimates respiratory muscle strength.
- It may be worth including reflex cough flow as an outcome. This is because reflex cough is more ecologically valid than voluntary cough.
- In the sample size calculation please provide the effect sizes used and reported in the studies cited by the authors. The sample size calculation requires more details. What study design the authors are applying the estimated effect sizes? Please note that an effect size for a two-way anova analysis is different than that for two mean comparison test, pre and post comparison, etcetera. Please also indicate the estimated error.
- I suggest providing more details about how the authors will apply the intention to treat principle.
- Please consider the anova design (two groupsXthree time points) plus group and time effect for sample size calculation.
- The Friedman test does not follow after two-way anova for post hoc comparisons. Please modify.
- In the method section of the abstract. Please note that by definition a randomized control trial is prospective. Please avoid redundancies.
