# Supplementary material for: Effects of high-intensity respiratory muscle training on respiratory muscle strength in individuals with Parkinson’s disease: Protocol of a randomized clinical trial
Source: PLoS One. 2023 Sep 8;18(9):e0291051. doi: 10.1371/journal.pone.0291051 (PMC10490961; doi:10.1371/journal.pone.0291051)
Supplement: Attachment — Submitted filename: Response to Reviewers.docx [file pone.0291051.s007.docx]

**RESPONSE TO THE editor**

**Ref:** PONE-D-23-05779

**Title:** “Effects of high-intensity respiratory muscle training on respiratory muscle strength in individuals with Parkinson's disease: protocol of a randomized clinical trial.”

**Review Comments to the Author**

We would like to thank you for the positive considerations regarding our manuscript. You provided excellent suggestions that helped to improve our work. All your comments, suggestions, and corrections were carefully taken into consideration and point-by-point responses are included below.

**Reviewer #3: Minor revisions:**

**1- Line 321: Indicate the statistical testing method(s) which attain 90% power.**

Power is the probability that a test will lead to rejection of the null hypothesis, or the probability of reaching statistical significance (Portney & Watkins, 2015). Power can be analyzed to estimate sample size or determine the probability that a Type II error has been made when a study results in a non-significant finding (Portney & Watkins, 2015). Researchers can estimate sample size by determining a significance level, effect size, and power. This is called an a priori power analysis (Portney & Watkins, 2015), which was used in the present protocol.

Two-way ANOVA is the statistical test planned to be used to analyze the data of the randomized controlled trial. The power can be determined for each of the main effects and interaction effects of the two-way ANOVA (Portney & Watkins, 2015). According to the tables provided by Portney & Watkins (2015) for analysis of variance, the power is approximately 90% for an effect size of 0.5, considering an α = 0.05 and a sample size of 30 individuals (Portney & Watkins, 2015). This power was used aiming at a more conservative estimate since larger samples have greater statistical power (Portney & Watkins, 2015).

As suggested, this information has been added to the manuscript, as follows:

Line 230-233: *“For analysis of variance, the power is approximately 90% for an effect size of 0.5, considering an α = 0.05 and a sample size of 30 individuals [18]. This power was used aiming at a more conservative estimate since larger samples have greater statistical power [18].”*

**2- Line 347: State the statistical method that will be used for the post-hoc tests.**

In most post hoc analyses, the specific comparisons of interest are decided after the analysis of variance has been completed and are considered unplanned comparisons (Portney, 2020). The choice of the post-hoc tests must be done considering some characteristics such as equal or unequal sample size and the assumption (or not) of equal variances. Fisher’s Least Significant Difference (LSD), Tukey’s Honestly Significant Difference (HSD) or Bonferroni are some of the statistical methods that can be used for the post-hoc tests (Portney, 2020). However, only after the analysis of variance has been completed, it will be possible to correctly select the post-hoc test.

As suggested, the manuscript text was revised, as follows:

Line 248-251: *“Two-way ANOVA with repeated measures (2*3) will be used to evaluate the differences between groups, considering the time factor (baseline, post-intervention, and 12-week follow-up), followed by post hoc test selected based on final sample size and in the assumption of equal of variance [18].”*

**3- Line 345: Clarify if the primary test of interest is the interaction effect of group by time.**

Between-group statistical comparison considering the interaction effect of group by time (baseline, post-intervention, and 12-week follow-up) is the primary test of interest.

This information is described in text, as follows:

Line 248-251: *“Two-way ANOVA with repeated measures (2*3) will be used to evaluate the differences between groups, considering the time factor (baseline, post-intervention, and 12-week follow-up), followed by post hoc test selected based on final sample size and in the assumption of equal of variance [18].”*

**4- Line 336: The following statement is unclear. Does this imply that the distribution of continuous variables will be checked for normality? If so, state the method(s) that will used. "The normality of data distribution will be for all continuous numeric variables."**

As suggested, the text of the manuscript was revised, as follows:

Line 337-338: *“The distribution of continuous variables will be checked for normality using the Shapiro-Wilk test.”*
